# Genetic diversity of drug resistant *Mycobacterium Tuberculosis* in local area of Southwest China: a retrospective study

**DOI:** 10.1186/s12879-018-3503-0

**Published:** 2018-11-14

**Authors:** Tao Shi, Tongxin Li, Jungang Li, Jing Wang, Zehua Zhang

**Affiliations:** 1 Department of Orthopedics, The Third Affiliated Hospital of Chongqing Medical University (Gener Hospital), No. 1, Shuanghu Branch Road, Yubei District, Chongqing, 401120 China; 2 Department of Clinical Laboratory, Public Health Medical Center, No. 109, Baoyu Road, Shapingba District, Chongqing, 400036 China; 3 Department of Orthopedics, Southwest Hospital, Third Military Medical University, No. 30, Gaotanyan Main Street, Shapingba District, Chongqing, 400038 China

**Keywords:** *Mycobacterium tuberculosis*, Drug resistance, Genetic diversity, Transmission

## Abstract

**Background:**

By 2014 although tuberculosis (TB) incidence had fallen by an average of 1.5% per year since 2000 and was 18% lower than the level of in 2000, 1.5 million people died for TB in that year. One of reason was that drug resistant *Mycobacterium tuberculosis* (DRTB) spread. This study aims to determine drug resistant characteristics and genotype of DRTB that isolated from patients in a tuberculosis referral hospital of southwest China.

**Methods:**

Five hundred thirty-eight drug resistant tuberculosis samples were collected from July 2013 to March 2015. All the isolates were identified by genomic deletions in region of difference 105 (RD105) and genotyped by mycobacterial interspersed repetitive unit-variable number tandem repeat typing (MIRU-VNTR). Polymorphism and cluster analysis of each locus was carried out using Bionumerics Version 3.0 and phyloviz software.

**Results:**

Five hundred thirty-eight TB strains included 503 *Mycobacterium tuberculosis* (MTB) isolates and 35 non *Mycobacterium tuberculosis* (NMTB) isolates. Of 503 isolates Beijing family type was 447 (88.9%, 447/503) and non-Beijing family type was 56 (11.1%, 56/503). Five hundred three DRTB isolates were divided into 345 genotypes, of which 265 isolates were single genotype and the remaining 238 strains were classified into 80 clusters with cluster rate of 47.3% and cluster ratio of 31.4%. Sixty-nine clusters belonged to Beijing family with cluster rate was 48.3% and clustering ratio was 32.9%. The non - Beijing family had 11 clusters with a cluster rate of 39.3% and the clustering ratio of 19.6%. Beijing genotype had a significant correlation with the age (*P* < 0.05), the retreatment patients (P < 0.05) and the city of Chongqing (P < 0.05), not with gender (*P* > 0.05). In the 9 Beijing genotype clusters each cluster contained some patients who lived in the same region.

**Conclusions:**

Beijing genotype was the predominant in the patients with DRTB in our hospital. In Chongqing retreatment patients with Beijing genotype MTB may be patient with DRTB. Drug resistance test (DST), regular medication and strict follow-up are very important for patients with Beijing genotype MTB. In Chongqing control and treatment of DRTB should be paid attention. Transmission and relations of patients with DRTB need to be further research.

## Background

With the development of tuberculosis diagnosis and treatment methods, the prevention and control of tuberculosis has made great progress. In 2014 tuberculosis (TB) incidence had fallen by an average of 1.5% per year since 2000 and was 18% lower than the level of 2000 according to the “Global Tuberculosis Report 2015” from the World Health Organization (WHO) [1]. Unfortunately about 3.3% of new cases and 20% of previously treated cases were drug resistant *Mycobacterium tuberculosis* (DRTB) [1]. In 2014 about approximately 1.5 million people died of DRTB [1].

China is one of the world’s 22 high tuberculosis burden countries and faces the challenge of DRTB, which accounted for 22 to 30% of all cases of tuberculosis [1]. It is unknown whether the spread of DRTB originates from acquire resistance or primary resistance, especially multidrug-resistant tuberculosis (MDR-TB) and extensive drug resistance tuberculosis (XDR-TB). MDR-TB is defined as resistance to at least isoniazid and rifampin, while XDR-TB is defined as having resistance to rifampin and isoniazid as well as any member of the quinolone family and at least one of the remaining second-line anti-TB injectable drugs. The traditional epidemiology investigation lack optimal way to gain answer about this problem.

Early molecular epidemiological tools had provided a reliable way to investigate molecular evolution over shorter and longer periods of time [2–5]. Although IS*6110*-restriction fragment length polymorphism DNA fingerprinting had been the genotyping technique used most widely for MTB and was considered a “gold standard”, it is no longer widely used due to time-consuming, technically demanding and requirement of large quantities of high-quality DNA [6]. According to comparison results of different ways, spoligotyping or RD105 was a reliable standard for identifying strains as belonging to the Beijing family because it is simple, highly reproducible and applicable to a digital format and mycobacterial interspersed repetitive unit–variable number tandem repeat typing (MIRU-VNTR) was the most reliable method for the genetic differentiation of MTB isolates because the discriminatory power of this method can be comparable to that of IS*6110* typing [7–9].

Chongqing is the largest municipality located in southwestern China and a city of high incidence of tuberculosis. An epidemiological study demonstrated that the rates of primary and acquired MDR-TB were 3.8 and 26.9%, respectively [10]. Sichuan Province is an adjacent area at the northwest Chongqing and also a high incidence of tuberculosis [8]. Nevertheless; we still have no knowledge of the potential transmission profile of DRTB in these areas. In this study in order to determine drug resistant characteristics, genotype and spread of DRTB, we genotyped the DRTB isolates using RD105 and MIRU-VNTR. The relationship between the molecular characteristics and transmission of DRTB was also analyzed.

## Methods

### Bacterial strains and culture conditions

From July 2013 to March 2015 a total of 753 samples from The Public Health Medical Center and the 12th People’s Hospital of Chongqing were collected. Various *M. tuberculosis* culture and identification systems were used during the study period. The bacterial strains and culture conditions were the same as what Weng described [11]. The first was the BACTEC MGIT 960 system (Becton Dickinson, Sparks, Maryland, USA). Clinical specimens were processed, and the centrifuged sediment was inoculated onto Löwensteine-Jensen (LJ) medium (BBL; Becton Dickinson, Sparks, MD, USA) and Middlebrook 7H9 broth (BBL; Becton Dickinson). The cultures were incubated at 35 °C in 5% carbon dioxide for up to 8 weeks. Identification of *M. tuberculosis* was based on colony morphology and biochemical reactions (nitrate reduction and niacin test). Bacterial cells were isolated from LJ medium.

### Drug susceptibility testing

The isolates were determined by conventional proportional drug susceptibility test. The concentrations of drugs in media were as follows: isoniazid (INH) 0.2 μg/ml, rifampicin (RFP) 40 μg/ml, ethambutol (EMB) 2 μg/ml, streptomycin (SM) 4 μg/ml, amikacin (AMK) 30 μg/ml, capreomycin (Cm) 40 μg/ml, levofloxacin (Lofx) 2 μg/ml, protionamide (Pto) 40 μg/ml and dipasic (PAIN) 0.1 μg/ml [10]. A strain was declared resistant to a drug when the growth rate was > 1% compared with the control. MDR-TB strains were defined as those resistant to both isoniazid and rifampicin. In addition, isolates resistant to rifampicin and isoniazid as well as any member of the quinolone family and at least one of the remaining second-line anti-TB injectable drugs were defined as XDR-TB.

### Genomic DNA extraction

Genomic DNA was extracted from freshly cultured bacteria. Following centrifugation at 13000 rpm for 2 min, the bacterial cells were transferred to a microcentrifuge tube containing 500 ml Trisethylenediaminetetraacetic acid (TE) buffer. The supernatant was discarded and the pellet was resuspended in 500 ml TE buffer and heated in a 95 °C water bath for 1 h. The cellular debris was isolated by centrifugation at 13000 rpm for 5 min and the DNA in the supernatant was used for PCR amplification reactions.

### Genotyping

The identification of genomic deletions in region of difference 105 (RD105) was performed by PCR to distinguish Beijing type from non-Beijing type. Briefly, each PCR mixture was prepared in a volume of 20 μl containing 19 μl RD105 PCR Mix and 1 μl DNA template. The amplification cycle was 10 min at 95 °C followed by 25 cycles of 30 s at 94 °C, 30 s at 68 °C, and 3 min at 72 °C, with a final step for 7 min at 72 °C.

To identify a suitable MIRU-VNTR loci set for genotyping *M. tuberculosis* in this area, the number of tandem repeats was determined in 12 MIRU-VNTR genetic loci: four original MIRU-VNTR loci: MIRU-10, MIRU-26, MIRU-31, MIRU-40; one loci of exact tandem repeats (ETRs): ETR- F; two Mtub loci: Mtub-04, Mtub-21; five Queen’s University of Belfast (QUBs) loci: QUB-11b, − 18,-26, − 4156 and 1895. QUB-11b, QUB-18, QUB-26, QUB-4156, MIRU26, MIRU31, MIRU10, Mtub21 and Mtub04 locus of MTB isolates was amplified separately by PCR using specific primers. Briefly, 1 μl of DNA was added to 19 μl of reagent mix. The amplification parameters consisted of 10 min at 95 °C, followed by 30 cycles of 30 s at 94 °C, 30 s at 58 °C, and 90 s at 72 °C, with a final extension at 72 °C for 7 min. QUB-1895, MIRU40 and ETR-F locus of MTB isolates was amplified separately by PCR using specific primers. Briefly, 1 μl of DNA was added to 19 μl of reagent mix. The amplification parameters consisted of 10 min at 95 °C, followed by 30 cycles of 30 s at 94 °C, 30 s at 64 °C, and 90 s at 72 °C, with a final extension at 72 °C for 7 min. The PCR products were electrophoresed on a 1% agarose gel. The H37Rv strain was assayed in the same manner as a control. The Hunter–Gaston discriminatory index (HGDI) was used to evaluate the discriminatory power of the MIRU-VNTR loci. BioNumerics (version 5.0, Applied Maths, Sint-Martens-Latem, Belgium) was used to construct the Minimal Spanning Trees (MSTs) based on VNTR data. A dendrogram was constructed based on the chi square test and the software package MEGA (version 6.0).

### Statistical analysis

All data were presented as mean ± standard deviation (SD) or frequency. Statistical analysis for possible significant association between the different symptoms and different genotype *M. tuberculosis* was performed using Chi-square test. All tests were set as two sides and a *P* value of < 0.05 was considered statistical significant.

## Results

In the 753 samples there were 215 (28.6%) negative culture samples and 538 (71.4%) positive culture samples that included 503 *Mycobacterium tuberculosis (*MTB) isolates and 35 non *Mycobacterium tuberculosis* (NMTB) isolates.

### Demography

The mean age of 503 patients including 189 female and 314 male was 38.9 ± 14.9 years old (16–78 years old). There were 201 new patients and 302 previously treated patients. Table 1 displayed distribution of patients in different regions. There are 19 districts and 19 counties in Chongqing and 21 cities in Sichuan Province. Most patients lived in Chongqing regions or near the Chongqing and few patients lived out of Chongqing, but all the patients lived in Chongqing when they suffered from DRTB. Patients from Chongqing accounted for 82.1, 16.1% from Sichuan Province and 1.8% from other regions.

**Table 1:** Distribution of 503 patients in different regions (*n* = 503)

Chongqing (*n* = 413)						Sichuan Province (*n* = 81)		Other regions (*n* = 9)	
Pengshui	62	Tongliang	10	Dazu	2	Dazhou	44	Tibet	1
Shapingba	33	Yuzhong	10	Fengjie	2	Guangan	23	Jilin Province	1
Banan	29	Wulong	9	Youyang	2	Ziyang	4	Hubei Province	1
Yubei	25	Dadukou	9	Yunyang	2	Yibin	3	Heilongjiang Province	1
Fengdu	21	Yongchuan	6	Wushan	2	Guangyuan	2	Guizhou Dejiang	1
Nanan	20	Jiangjin	6	Tongnan	2	Luzhou	2	Guizhou Fenggang	1
Beibei	20	Nanchuan	6	Rongchang	1	Neijiang	2	Guizhou Qianxi	1
Changshou	19	Bishan	5	Shizhu	1	Chengdu	1	Guizhou Tongren	1
Zhong	16	Kai	5	Wanzhou	1			Guizhou Yinjiang	1
Fuling	15	Qijiang	5	Xiushan	1				
Hechuan	15	Liangping	5	Qiangjiang	1				
Jiulongpo	14	Wansheng	4						
Dianjiang	13	Wuxi	4						

### Characteristics of DRTB

**Fig. 1:**
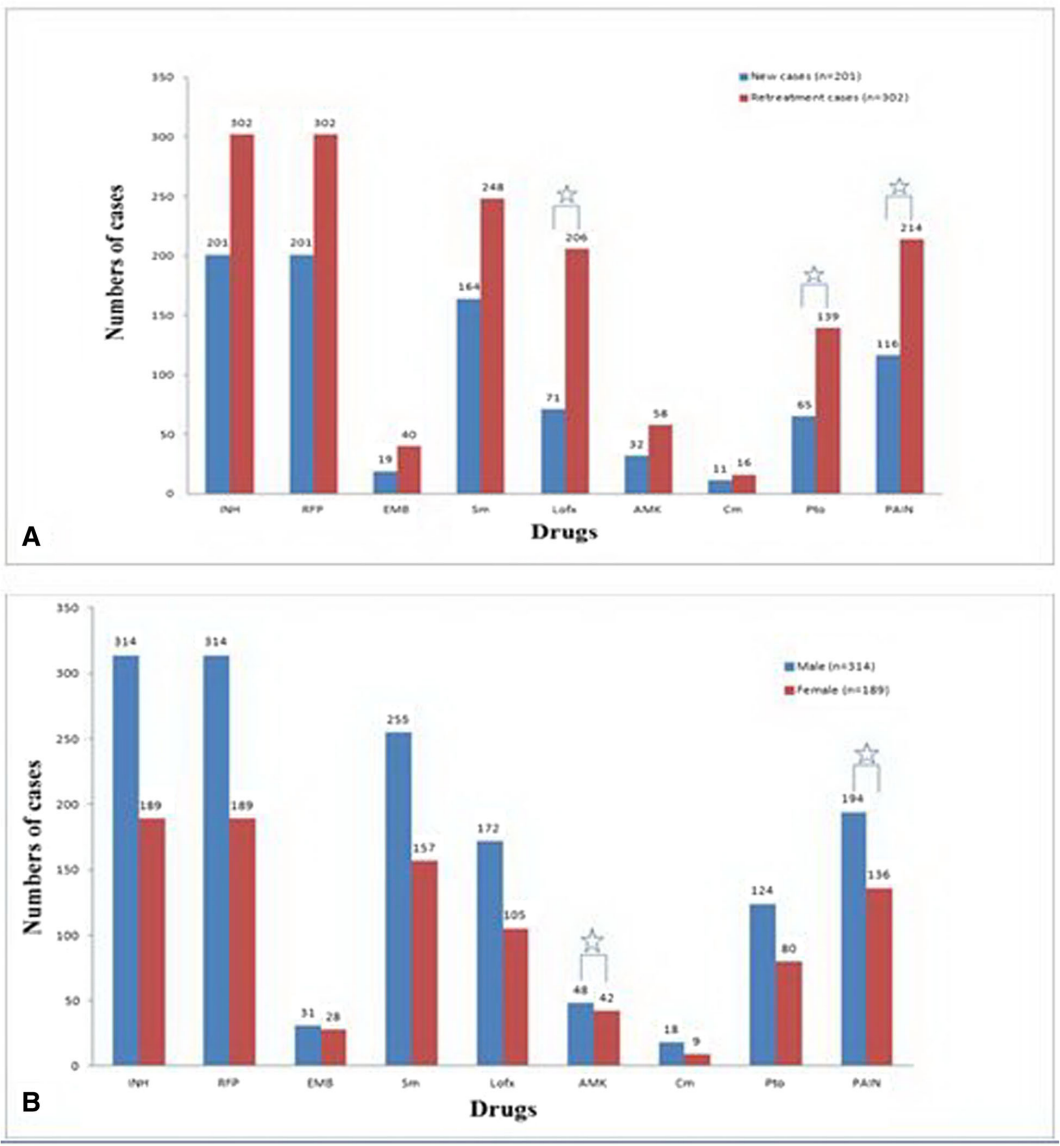
**a** Distribution of the new cases and retreatment cases in the different anti-TB drugs. **b** Distribution of femal and male patients in the different anti-TB drugs. ✩ Means *P* < 0.05

All the 503 isolates were MDR-TB. There were 75 (14.9%) XDR-TB in the 503 isolates. Of 503 patients there were some special varieties of resistant drugs. There were 34(6.8%) patients with only resistance to INH and RFP including 27(5.4%) new and 7(1.4%) retreatment patients; 61(12.1%) only resistance to first-line drug including 43(8.5%) new and 18(3.6%) retreatment patients; 54(10.7%) resistance to INH, RFP and any second-line drug including 15(3.0%) new and 39(7.7%) retreatment patients; 17(3.4%) resistance to all test drugs including 5(1.0%) new and 12(2.4%) retreatment patients. The numbers of new and retreatment of patients resistant to Lofx, Pto and PAIN were a significant statistical difference, retreatment patients were more obviously resistant to the above three drugs than new cases (Fig. 1a). Male patients of resistant to AMK and PAIN had a significant statistical difference with female patients; the male was more obviously resistant than the female resistant to AMK and PAIN (Fig. 1b). Resistance to PAIN was more common than the other anti-TB drugs in the retreatment patients and male patients.

### The identification of genomic RD105

The collection of 503 DRTB isolates was analyzed by RD105 in this study. 447 (88.9%) isolates belonged to the Beijing genotype, while 56 (11.1%) were from non-Beijing families, demonstrating that Beijing is the predominant genotype.

### MIRU-VNTR profiles and genotypes

Five hundred three strains were divided into 345 genotypes that included 265 strains were a single genotype and the remaining 230 strains were classified into 80 clusters (2–24 isolates per cluster). The cluster rate was 47.3% and the cluster ratio was 31.4%. Four hundred forty-seven Beijing genotype isolates were clustered into 69 genotypes and 56 non-Beijing genotype isolates were clustered into 11 genotypes. The cumulative clustering rates of Beijing genotype and non-Beijing genotype strains were 48.3 and 39.3%, respectively. The both clustering ratio were 32.9 and 19.6%, respectively. The non-Beijing genotype isolates came from the newly diagnosed patients.

**Fig. 2:**
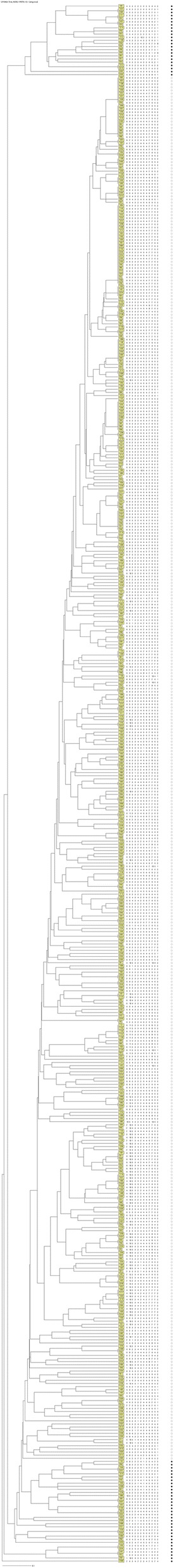
Dendrogram of 503 DRTB isolates. The phylogenetic tree was generated from the MIRU-VNTR profile

**Fig. 3:**
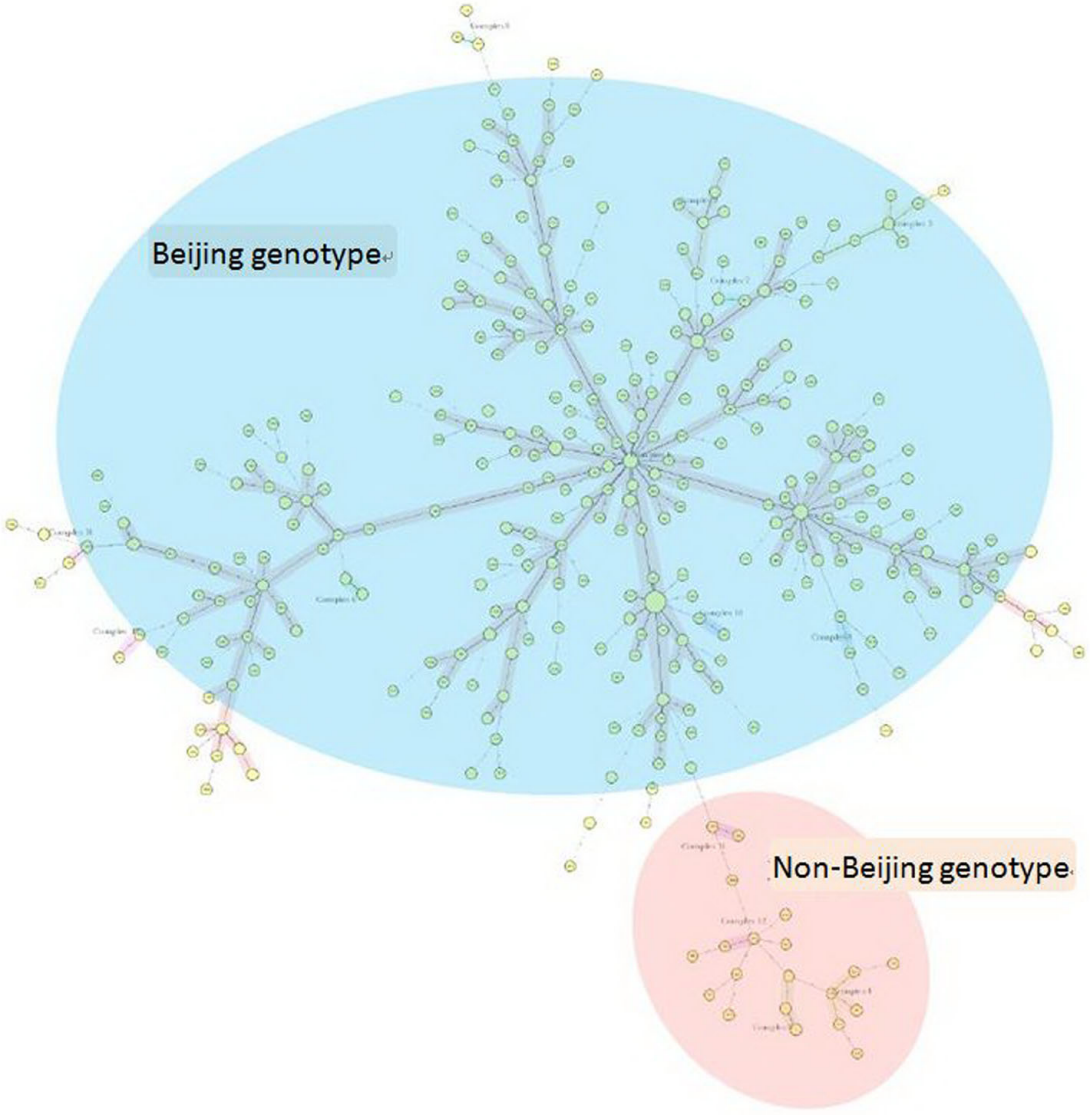
Minimum spanning tree (MST) of 503 DRTB isolates. The MST was generated from the MIRU-VNTR profile

The details of 80 clusters were displayed in the Table 2 and Fig. 2. Figure 3 displayed the minimum spanning tree of 503 DRTB isolates according to MIRU-VNTR results.

**Table 2:** Details of 80 clusters

Genotype	No. of cluster	No. of strains in each cluster
Beijing-type		
	1	26
	1	11
	1	10
	1	9
	1	8
	1	7
	4	4
	9	3
	50	2
Non-Beijing-type		
	11	2

HGDI showed that the 12-site combination had a total resolution index of 0.99. The allelic diversity of each MIRU-VNTR locus was evaluated using the HGDI (Table 3). Overall, the HGDI of four loci (QUB18, Mtub21, QUB26 and QUB11b) exceeded 0.6, classified as highly discriminating loci. The six loci (QUB1895, QUB4156, MIRU26, MIRU31, MIRU40 and Mtub04) showed moderate discrimination (0.3 < HGDI< 0.6). The MIRU10 and ERT-F showed low discrimination loci. A dendrogram was generated based on the genotypes of the 503 isolates using 12 loci.

**Table 3:** Allelic diversity of 12 different MIRU-VNTR loci among Mycobacterium tuberculosis strains

	Locus	HGDI
1	QUB-11b	0.62
2	QUB-18	0.65
3	QUB-26	0.66
4	QUB-4156	0.37
5	QUB-1895	0.37
6	MIRU26	0.57
7	MIRU31	0.47
8	MIRU10	0.26
9	MIRU40	0.44
10	Mtub21	0.62
11	Mtub04	0.55
12	ETR-F	0.26

### Comparison between demographic characteristics and DRTB

Clinical factors, including age, gender and treatment history were analyzed between the Beijing and non-Beijing family (Table 4). Beijing genotype had a significant correlation with the age (*P* < 0.05), the retreatment patients (P < 0.05) and Chongqing (P < 0.05), not with gender (*P* > 0.05). In particular, isolates from patients with retreatment were all Beijing genotype, and there was no statistical result.

**Table 4:** Clinical characteristics of the Beijing and non-Beijing family strains

Characteristics	Total	Beijing family	Non-Beijing family	χ^2^	*P*-value
Age, years					
≤30	153	150	3	784.00	< 0.05
30–60	306	279	27		
≥60	44	41	3		
Gender					
Male	314	289	25	2.68	> 0.05
Female	189	181	8		
Treatment history					
New cases	201	168	33	214.40	< 0.05
Retreatment	302	302	0		
Regions					
Chongqing	413	402	11	25.40	< 0.05
Sichuan Province	81	78	3		
Other regions	9	6	3		

### Distribution of DRTB

All patients lived in the above regions. The living regions of patients carrying clustering isolates were analyzed. Although all the patients carrying non-Beijing genotype clustering isolates came from different regions of Chongqing, some patients carrying Beijing genotype clustering isolates came from the same regions of Chongqing. Table 5 displayed regional distribution of some patients with Beijing genotype. The patients of the remaining Beijing genotype clusters came from different regions of Chongqing. In each non-Beijing genotype cluster there were no patients who come from the same region. In our study Pengshui, Shapingba and Banan were three regions of high incidence in Chongqing and locate in the southeast Chongqing, while Dazhou and Guangan were two regions of high incidence in Sichuan Province and locate in northeast Sichuan Province (Table 1).

**Table 5:** Regional Distribution of some patients with Beijing genotype

No. of strains in one cluster	Region	No. of patients	No. of strains in one cluster	Region	No. of patients
26	Pengshui	7	8	Fengjie	2
	Beibei	2		Yubei	2
	Zhong	2		others	4
	others	15	7	Guangan	3
11	Nanan	2		others	4
	Banan	2	4	Zhong	3
	others	7		others	1
10	Pengshui	2	3	Shapingba	2
	others	8		others	1
9	Beibei	3	3	Jiangbei	2
	others	6		others	1

“others” mean “the rest patients came from different regions and only one patient in one region”

## Discussion

The Beijing genotype of *Mycobacterium tuberculosis* was first discovered by Soolingen in 1995 [12]. Since then, several studies have reported that Beijing genotype MTB is the main pathogen type of TB and DRTB patients [13–18]. In our study, a large number of patients with multiple MDR patients in Chongqing were collected. The basic types of DRTB were analyzed systematically. The results showed that DRTB in this region was mainly Beijing family type, accounting for 93.4%, which is similar to most of the current DRTB molecular type of research reports but not exactly the same. The results of the national tuberculosis drug resistance baseline analysis showed that the DR ratio of Beijing genotype strains was 63.97% in China and 59.97% in the southwest China [19], while our study showed the DR ratio of Beijing genotype strains was 88.9% that was significantly higher than the levels of nationwide and southwest China. This suggests that the prevalence of the Beijing genotype family in Chongqing may result in the spread of DRTB.

Anti-TB chemotherapy is the cornerstone of treatment of patients with MTB [20]. Unfortunately, evolution of TB leads to be resistant to anti-TB drugs. DRTB is a big challenge to anti-TB treatment. Many researchers studied the DR characteristics of DRTB. Fox example, according to Kapil’s research the DR ratio of INH, RFP and SM were 92.7, 81.9 and 69.3%, respectively [21]. Another article showed SM and EMB were most common resistant drugs [10]. In this study INH, RFP and SM were three most common first-line anti-TB resistant drugs and Lofx and PAIN were common second-line anti-TB resistant drugs. In addition DR ratio of Lofx, Pto and PAIN in the retreatment patients was higher than in the new case and resistance to AMK and PAIN was more common in male patients than female. These results may be helpful for treatment of patients with DRTB in southwest China. Different studies have shown the DR ration of different anti-TB drugs varied by regions.

In this study, 503 cases of DRTB strains were divided into 80 clusters and 345 genotypes with a clustering rate of 47.3%. Two hundred sixty-five patients were isolated as a single genotype, accounting for 52.7% of all patients (265/503), which may be considered to be independent isolates and no mutual transmission between patients, but rather independent infection or endogenous recurrent disease. The remaining 238 strains were classified in to 80 clusters, that the largest cluster of which contained 26 Beijing genotype isolates, including 22 isolates from the Chongqing Municipal Public Health Medical Treatment Center and 4 isolates from the Twelfth People’s Hospital of Chongqing. By analyzing the redidential regions of patients in the clusters that contained ≥3 isolates, we found some isolates transmitted within one region and had tendency transmitting to an adjacent area. There was a study about MDR transmitting across countries [22]. All these researches suggested the DRTB were transmitting more and more widely. Additional, each cluster of DRTB highly suggested that these isolates belonged to their respective groups and in each group isolates had a certain relationship according to results of MIRU-VNTR. Cross analysis results of MIRU-VNTR and living locations of patients suggested that patients with DRTB were cross-infection, that is, these patients with DRTB are primary resistant, rather than due to withdrawal and relapse. Continue to track these patients to determine the cause of resistance, such as whether patients with the same genotype are relative, contact history and living in the same community, which is the focus of our follow-up study.

MTB genotyping can explain the epidemiology, infection, pathogenesis and DR of MTB from the molecular level and it is important in TB epidemiological investigation, surveillance and transmission source discovery and transmission pathways. MIRU-VNTR was classified according to the difference of the number of copies between different strains. The method was simple, the result was digitized and the resolution was high, which was convenient for comparison between different laboratories. Compared to IS*6110*-RFLP which used to be the “gold standard” for DNA fingerprints of MTB, MIRU-VNTR has obviously advantages. Using the MIRU-VNTR 191 Beijing family-type MTB were divided into 110 unique genotype and 27 clusters in China [8] and in Sichuan 191 Beijing Mycobacterium tuberculosis has 65 unique genotypes and 8 clusters [23]. In addition, Chinese Center for Disease Control and Prevention analyzed 4017 MTB isolates from 31 different provinces that were divided into 161 clusters and 407 isolates using spoligotyping and MIRU-VNTR [24]. According to the results of a study comparing different methods RD105 or spoligotyping is a reliable method for identifying whether the strain belongs to the Beijing cluster, although the identification ability may be not comparable to that of IS*6110*-RFLP. MIRU-VNTR was the most reliable method for the genetic differentiation of MTB isolates because the discriminatory power of this method is also comparable to that of IS*6110* typing [7–9]. The different research purposes of MTB molecular epidemiology study should select the appropriate technological choice ways based on different research purposes, or combined variety of ways in order to improve work efficiency and accuracy of the results.

The discrimination of the MIRU-VNTR depends on the resolution of the site used and the VNTR loci should be selected according to the genetic polymorphism of MTB in different regions. The reports had displayed that QUB18, Mtub21, QUB26, QUB11b, QUB11a, and MIRU26 were highly discriminating loci in Chongqing [25] and Mtub04, Mtub21, Mtub39, QUB26, QUB11b, MIRU10, MIRU26, MIRU39, MIRU40, ETRA and ETRE were highly discriminating loci in Sichuan province [26]. In this study, VNTR genotyping was carried out at 12 sites, which were representative of both international and domestic locations. By analyzing the allelic polymorphism of the isolates, the HGDI of polymorphism of 12 loci was between 0.29 and 0.67. The results showed that the seven loci including QUB-11b, QUB-18, QUB-26, MIRU26, MIRU31, Mtub21 and Mtub04 had high discrimination in MTB in Chongqing area. But the discrimination of MIRU10 in Chongqing is lower than that of Sichuan province. Compared with the typing of Yongchuan in Chongqing the Mtub04 loci selected in this study have a relatively high resolution [10], while MIRU10 and ETR-F are relatively low, which may be related to the size of the selected study sample range. Therefore, in the future the classification work will select high polymorphic combination of sites including QUB-11b, QUB-18, QUB-26, MIRU26, MIRU31, Mtub21 and Mtub04 that is a great help for increasing classification efficiency.

## Conclusions

Beijing genotype was the predominant in the patients with DRTB in our hospital. In Chongqing retreatment patients with Beijing genotype MTB may be patient with DRTB. DST, regular medication and strict follow-up are very important for patients with Beijing genotype MTB. In Chongqing control and treatment of DRTB should be paid attention. Transmission and relations of patients with DRTB need to be further research.

## Abbreviations

AMK: Amikacin
Cm: Capreomycin
DNA: Deoxyribonucleic acid
DRTB: Drug resistant Mycobacterium tuberculosis
DST: Drug susceptibility testing
EMB: Ethambutol
ETRs: Exact tandem repeats
HGDI: Hunter–Gaston discriminatory index
INH: Isoniazid
Lofx: Levofloxacin
MDR-TB: Multi-drug resistance Tuberculosis
MGIT: Mycobacterium Growth Indicator Tube
MIRU-VNTR: Mycobacterial interspersed repetitive unit-variable number tandem repeat typing
MSTs: Minimal Spanning Trees
NMTB: Non Mycobacterium tuberculosis
PAIN: Dipasic
PCR: Polymerase chain reaction
Pto: Protionamide
QUB: Queen’s University of Belfast
RD105: Region of difference 105
RFP: rifampicin
SM: Streptomycin
TB: Tuberculosis
WHO: World Health Organization
XDR-TB: Extensive drug resistance tuberculosis
